# Alpha-mannosidosis

**DOI:** 10.1186/1750-1172-3-21

**Published:** 2008-07-23

**Authors:** Dag Malm, Øivind Nilssen

**Affiliations:** 1 Department of Gastroenterology, University Hospital of North Norway, NO-9038, Norway; 2 Department of Medical Genetics, University Hospital of North Norway, NO-9038, Norway; 3 Institute of Clinical Medicine, University of Tromsø, NO-9037, Norway

## Abstract

Alpha-mannosidosis is an inherited lysosomal storage disorder characterized by immune deficiency, facial and skeletal abnormalities, hearing impairment, and intellectual disability. It occurs in approximately 1 of 500,000 live births. The children are often born apparently normal, and their condition worsens progressively. Some children are born with ankle equinus or develop hydrocephalus in the first year of life. Main features are immune deficiency (manifested by recurrent infections, especially in the first decade of life), skeletal abnormalities (mild-to-moderate dysostosis multiplex, scoliosis and deformation of the sternum), hearing impairment (moderate-to-severe sensorineural hearing loss), gradual impairment of mental functions and speech, and often, periods of psychosis. Associated motor function disturbances include muscular weakness, joint abnormalities and ataxia. The facial trait include large head with prominent forehead, rounded eyebrows, flattened nasal bridge, macroglossia, widely spaced teeth, and prognathism. Slight strabismus is common. The clinical variability is significant, representing a continuum in severity. The disorder is caused by lysosomal alpha-mannosidase deficiency. Alpha-mannosidosis is inherited in an autosomal recessive fashion and is caused by mutations in the *MAN2B1 *gene located on chromosome 19 (19 p13.2-q12). Diagnosis is made by measuring acid alpha-mannosidase activity in leukocytes or other nucleated cells and can be confirmed by genetic testing. Elevated urinary secretion of mannose-rich oligosaccharides is suggestive, but not diagnostic. Differential diagnoses are mainly the other lysosomal storage diseases like the mucopolysaccharidoses. Genetic counseling should be given to explain the nature of the disease and to detect carriers. Antenatal diagnosis is possible, based on both biochemical and genetic methods. The management should be pro-active, preventing complications and treating manifestations. Infections must be treated frequently. Otolaryngological treatment of fluid in the middle ear is often required and use of hearing aids is invariably required. Early educational intervention for development of social skills is needed and physiotherapy is important to improve bodily function. Orthopedic surgery may be necessary. The long-term prognosis is poor. There is an insidiously slow progression of neuromuscular and skeletal deterioration over several decades, making most patients wheel-chair dependent. No patients manage to be completely socially independent. Many patients are over 50 years of age.

## Disease name and synonyms

**α**-Mannosidosis, Lysosomal **α**-D-Mannosidase Deficiency, **α**-Mannosidase B Deficiency. OMIM 248500.

## History

Hurler-like syndrome had been known for a number of years when the Swedish physician Öckerman in Lund described a 4 year old boy with a Hurler-like phenotype in 1967 [1]. He died at the age of 4 from pneumonia, and in his tissues appeared large amounts of oligosaccharide material with the dominance of mannose. Therefore, the term "Mannosidosis" was suggested as the name of the disorder [2]. In 1977 Loeb described an atypical form of mucopolysaccharidosis which later turned out to be **α**-mannosidosis [3].

In 1978, Gideon Bach described two Palestinian siblings with a mild clinical phenotype and residual mannosidase activity, which increased by 40% with the addition of zinc (Zn^++^) to cell extracts of both patients and control subjects [4]. It took 25 years before the molecular mechanism of this observation could be explained [5]. Meanwhile, this observation led to many therapeutic attempts with zinc substitution in cattle [6] and man [7], which all proved futile.

Since these early clinical descriptions, many research groups have contributed to the characterization of the enzyme and the corresponding gene in several species such as human, cow, cat, mouse and guinea pig. Furthermore, underlying genetic, biochemical and physiological mechanisms of the disease have been explored, and additional clinical aspects of the disease such as immunodeficiency and psychiatric complications of the disease have been described (see below). Subsequently, two EU Research Consortiums, EURAMAN 2002–2005 (A systematic and multidisciplinary approach towards understanding and therapy of the inborn lysosomal storage disease **α**-mannosidosis), and HUE-MAN 2006–2009 (Towards the Development of an Effective Enzyme Replacement Therapy for Human **α**-Mannosidosis) were established [8].

## Definition and diagnosis criteria

Alpha-mannosidosis is a genetic disorder of metabolism characterized by immune deficiency, facial and skeletal abnormalities, hearing impairment, and mental retardation. The disorder is caused by lysosomal **α**-mannosidase deficiency and is inherited in an autosomal recessive fashion.

Elevated urinary secretion of mannose-rich oligosaccharides is suggestive, but not diagnostic for **α**-mannosidosis. Diagnosis is made by measuring acid **α**-mannosidase activity in leukocytes or other nucleated cells, *e.g. *fibroblasts. Genetic diagnostics by mutation analysis is available from a few laboratories.

Alpha-mannosidosis has been described as two distinct phenotypes: one severe form with hepatomegaly and early death following severe infections (Type I), and a mild form with hearing loss, mental retardation, and survival into adulthood (Type II) [4,9,10].

However, when studying published cases, the patients present a continuum of clinical presentations, many of which probably can be influenced by background genetics or external factors like infectious diseases, educational opportunities, proactive initiatives, and quality of health services [11-13].

At present, three clinical types have been suggested [14-16]: Type 1: Mild form clinically recognized after 10 years of age, without skeletal abnormalities and very slow progression; Type 2: Moderate form, clinically recognized before 10 years of age, with skeletal abnormalities, and slow progression with development of ataxia at age 20–30; Type 3: Severe form, immediately recognized, with skeletal abnormalities, and obvious progression, leading to an early death from primary central nervous system (CNS) involvement or myopathy (Table 1). Most patients belong to clinical type 2.

**Table 1:** Clinical types of alpha-mannosidosis

**Type II**: Less severe, late onset form involving hearing loss, coarse face, mental retardation, and hepatosplenomegaly.	**Type I**: Severe infantile form which is fatal at <3–8 years of age.	
**Type 1**: Mild form clinically, recognized after 10 years of age, without skeletal abnormalities and very slow progression.	**Type 2**: Moderate form, clinically recognized before 10 years of age, with skeletal abnormalities, and slow progression with development of ataxia at age 20–30.	**Type 3**: Severe form, immediately recognized, with skeletal abnormalities, and obvious progression, leading to an early death from primary CNS involvement or myopathy.

Staging of disease severity of mannosidosis according to Desnick *et al.*, 1976 [9] (Upper panel) and according to Chester *et al.*, 1982 and Malm & Nilssen, 2006, [14,16] (Lower panel).

## Epidemiology

The prevalence of the disease is not precisely known. A study from Norway reported six (later eight) patients in a population of 4.5 millions [17]. This corresponds with a study from Australia, reporting a disease frequency of one in 500,000 live births [18]. Mannosidosis is expected to be found in any ethnic group anywhere in the world.

## Clinical description

Alpha-mannosidosis should be suspected in individuals with mental retardation, skeletal changes, hearing loss, and recurrent infections. The children are often born apparently normal, and their condition worsens progressively. Therefore, early diagnosis is important if bone marrow transplantation (BMT) is to be considered a therapeutic modality. Since inborn errors of lysosomal metabolism occur in approximately 1:5,000 live births, many have argued for screening of newborns for early diagnosis and initiation of treatment [18].

### Onset of symptoms

Early references have described that early psychomotor development appears normal, and that pathophysiology develops over time [4,19]. However, some children are born with ankle equinus or develop hydrocephalus in the first year of life [17,20].

In **α**-mannosidosis guinea pigs, long before the onset of obvious neurologic abnormalities at 2 months, cerebral pathophysiology like neuronal lysosomal vacuolation, and reduced myelination of white matter was observed. Thus, complex neuropathologic changes in **α**-mannosidosis guinea pigs are already present at birth, long before clinical changes are evident, and similar events are likely to occur in humans with this disorder [21].

### Associated facial features

**Figure 1:**
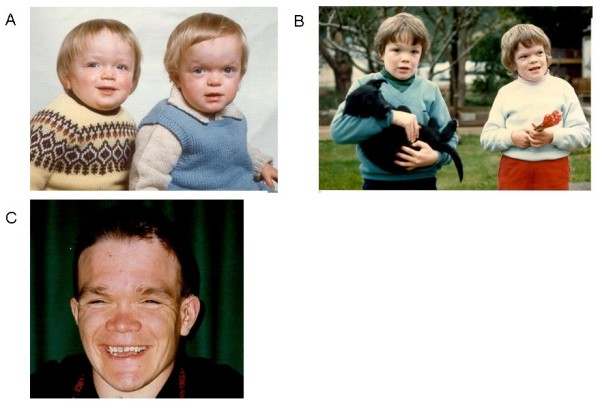
**Facial features in alpha-mannosidosis**. A. Twins aged 18 Months. Note enlarged head, short neck, rounded eyebrows, saddle nose, and prominent forehead. B. Same twins aged 8 years. Note slight muscular atrophy of the hands. C. Boy, aged 27. Note: Hearing aid.

Facial traits may be subtle, but independent of race and background genetics, all patients have some degree of coarse Hurler-like features. This is classically a large head with prominent forehead, rounded eyebrows, flattened nasal bridge, macroglossia, widely spaced teeth, and prognathism. The neck is usually short (Fig 1).

### Associated skeletal abnormalities

**Figure 2:**
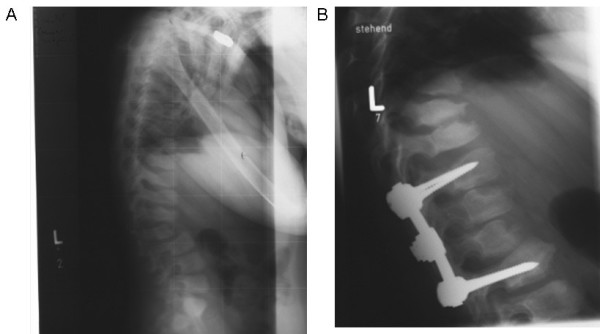
**Radiography of kyphoscoliosis in alpha-mannosidosis before and after orthopedic correction**. A. Kyphoscoliosis with skeletal abnormalities of all vertebrae. B. Orthopedic correction of kyphosis.

**Figure 3:**
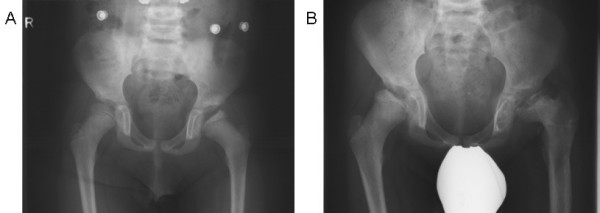
**Coxarthrosis in alpha-mannosidosis**. A. Coxarthrosis at 8 years of age. B. Progression of coxarthrosis at age 13.

According to Chester (1982), clinical or radiographic signs of mild-to-moderate dysostosis multiplex are present in 90% of the patients [14]. The most frequent abnormalities are scoliosis and deformation of the sternum [22] (Fig 2). These changes are present at birth. Genu valgus (knucle knee) is common and, like the same complication in Gaucher disease, may be treated with epiphyseal arthrodesis at a young age before the epiphyseal lineation of the knee is closed [23]. Over time, from the second till the fourth decade of life the patients may develop destructive polyarthropathy, especially coxarthrosis (Fig 3), but also gonarthrosis [24,25]. These are often so serious that orthopedic corrections are needed [26]. Patellar bilateral dislocation and severe synovial hypertrophy have also been described along with Charcot elbow and bilateral hip and elbow avascular necrosis in one patient [27].

### Associated hearing impairment

Moderate or severe sensorineural hearing loss seems inevitable [12,22,28,29]. It is regularly worsened by otitis or accumulation of fluid in the middle ear, adding a mechanical component to the hearing deficit [17].

### Associated ocular changes

Slight strabismus is common and also hyperopia more frequently than myopia [14,30]. Blurred discs [31], superficial corneal opacities, and lenticular changes seem to be rare but have been reported [4].

### Associated mental retardation

As soon as the age of the patients makes intelligence quotient (IQ) measurements feasible, almost all patients show some degree of mental retardation. In a clinical study that included eight patients the age at onset of symptoms varied from 6 months to 3 years [10]. The first symptom was usually delayed development of speech or motor or mental functions. All the patients were mildly or moderately mentally retarded with an IQ of 60–80, with a declining tendency over later decades. With their poor ability to speak combined with sensorineural hearing loss, patients score generally better in nonverbal tests [10]. Although some have suggested the disease stabilises in puberty [32], most follow-up observations suggest gradual impairment of mental and motor functions and speech with age. Longitudinal assessments of three brothers have been done by testing of general intelligence, language, visual spatial skills, and overall adaptive abilities over two years. During the follow-up, the cognitive findings showed that the patients manifested mild cognitive deficits. Cognitive deficits were generally uniform with no signs of progressive deterioration, except receptive language abilities [33]. Other patients are described as having profound retardation even at young age [19,34]. Again, there can be remarkable differences in mental functioning among siblings [28,35,36].

Patients learn to speak late, sometimes in the second decade of life. This impaired development of language with limited vocabularies and poor pronunciation might be explained by their congenital and/or later-onset hearing loss. Many patients learn to read, but have difficulties in understanding abstracts (Malm D: personal observation).

### Associated motor function disturbances

The development of motor functions in affected patients is generally slow, and the children appear clumsy. This is caused by a combination of factors like muscular weakness, joint abnormalities and ataxia due to cerebral atrophy and cerebral demyelination [37].

The impairment is by nature progressive, with gradual worsening in the second and third decade of life [10]. However, as for mental retardation, there is a considerable variation in the clinical progression and in a long term observation of two patients, no progression of neurological function has been noted [28], whereas some investigators have suggested that the disease progression is halted after puberty [32]. Additional studies on the natural course of the disease are needed.

### Associated psychiatric disease

In intellectually disabled patients, psychiatric symptoms may be overlooked. However, a systematic survey found psychiatric symptoms in more than 25% of adult patients with mannosidosis [38]. It typically presented in adolescence or early adulthood. In mentally retarded patients, psychiatric symptoms form part of a more diffuse clinical picture with systemic, cognitive or motor neurological signs. It can present with acute and recurrent attacks of confusion, sometimes with anxiety, depressions or, hallucinations. These might be associated with loss of function, like decreased appetite with severe weights loss or incontinence for urine and feces. The periods of psychosis usually last 3 to 12 weeks, followed by a long period of hypersomnia and sometimes loss of abilities, like difficulty speaking or inability to read. Search for organic causes has been negative [38]. As specific treatments should be more effective at the initial stage before the occurrence of irreversible neurological lesions, clinicians should be aware of atypical psychiatric symptoms in patients with inborn errors of metabolism [39].

### Associated immunodeficiency and autoimmunity

Mannosidosis patients suffer from recurrent infection, especially in the first decade of life. In one single patient, impaired leukocyte chemotaxis and reduced phagocytosis were found [9]. Malm and co-workers compared the humoral and cellular immunological functioning in six patients to that of six age- and sex-matched healthy controls [40]. They found that post-immunization levels of antibody were lower in patients, proving a decreased ability to produce specific antibodies after antigen presentation. More interestingly, there was a serum factor in patient plasma, inhibiting phagocytosis [40]. In mannosidosis, there are increased levels of oligosaccharides in plasma [41]. Oligomannosides with five and six mannose residues bind to interleukin-2 (IL-2) receptors disturbing the IL-2-dependent responses [42]. IL-2 activates T-, B-, and NK cells. It can therefore be speculated that blockage of this receptor is the mechanism causing the immune deficiency seen in mannosidosis.

The same mechanism could contribute to the increased prevalence of autoimmune disorders among mannosidosis patients ([13] and Malm D: personal observations). Interestingly, in a mice model, alpha-mannosidase II deficiency reduces complex-type N-glycan branching and induces an autoimmune disease similar to human systemic lupus erythematosus (SLE) with induction of antinuclear antibodies with reactivity towards histone, Sm antigen, and DNA [43].

### Associated renal and cardiac complications

End-stage kidney failure has been reported only once, where an Italian patient successfully received a kidney transplant [44]. In a study in mannosidosis mice, deposits of storage material in myocardium were reduced after enzyme replacement [45]. In some case descriptions, a murmur of the heart is mentioned, but so far, reports on manifest heart disease have not been reported.

## Molecular etiology

**Figure 4:**
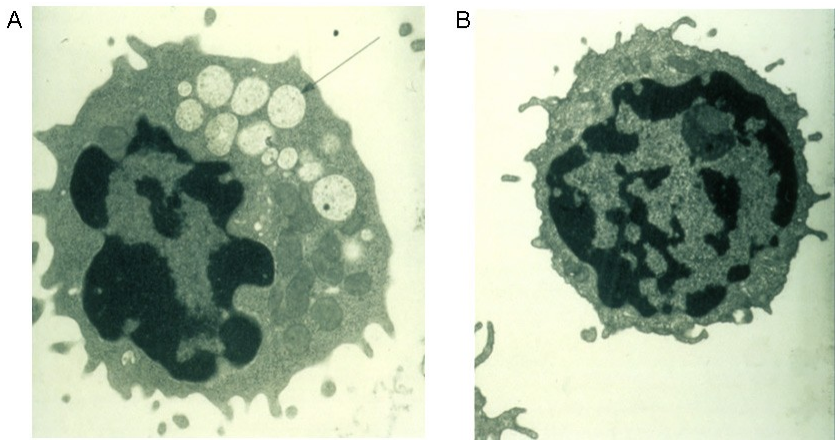
**Electron micrograph of a vacuolated lymphocyte**. Electron micrograph of a vacuolated lymphocyte from a mannosidosis patient (A) as compared to a lymphocyte from a normal control (B).

During normal turnover and catabolism glycoproteins are digested by proteinases and glycosidases within the lysosomes. These enzymes degrade glycoproteins into fragments small enough to be excreted or transported to the cytosol for reuse. Lack or deficiency of such hydrolases results in the multi-systemic accumulation of undigested material in the lysosomes. Consequently, the lysosomes swell resulting in severe impairment of cellular functions (Fig 4). However, the pathophysiology of lysosomal storage disorders is complex, and accumulation of storage material alone cannot fully explain the cause of disease.

Lysosomal **α**-mannosidase is an exoglycosidase which cleaves the **α**-mannosidic linkages during the ordered degradation of N-linked oligosaccharides. The enzyme has the capacity to cleave **α**(1 → 2), **α**(1 → 3) and **α**(1 → 6) mannosidic linkages found in high mannose and hybrid type glycans [46,47]. Deduced from the analyses of mannose rich fractions from urine of affected individuals the major lysosomal storage products are the oligosaccharides:

Man(**α**1 → 3)Man(**β**1 → 4)GlcNac, Man(**α**1 → 2)Man(**α**1 → 3)Man(**β**1 → 4)GlcNac and Man(**α**1 → 2)Man(**α**1 → 2)Man(**α**1 → 3)Man(**β**1 → 4)GlcNac [48].

However, several additional, but apparently less abundant urinary oligosaccharides have been identified [49], all of them with GlcNac at the reducing end (reviewed by [50]).

Alpha-mannosidosis is caused by mutations in the *MAN2B1 *(LAMAN) gene encoding lysosomal **α**-mannosidase. The *MAN2B1 *is located on chromosome 19 (19p13.2-p13.11) and is composed of 24 exons spanning 21.5 kb [51,52]. The *MAN2B1 *mRNA has the capacity to encode a polypeptide of 988 [53] or 1011 [54] amino acids depending on the start site of translation (Genebank accession numbers U68382/U68567 and U60266.1). As judged by Northern blot analyses, the level of *MAN2B1 *expression appears to be highest in lung [54], kidney, pancreas [53,54], and peripheral blood leukocyte [53]. In CNS the highest level of expression appears to be in corpus callosum and spinal cord, whereas considerably lower levels are observed in the larger structures, which include cerebellum, cerebral cortex, frontal and temporal lobes [54]. The significance (if any) of this variation is not clear.

**Figure 5:**
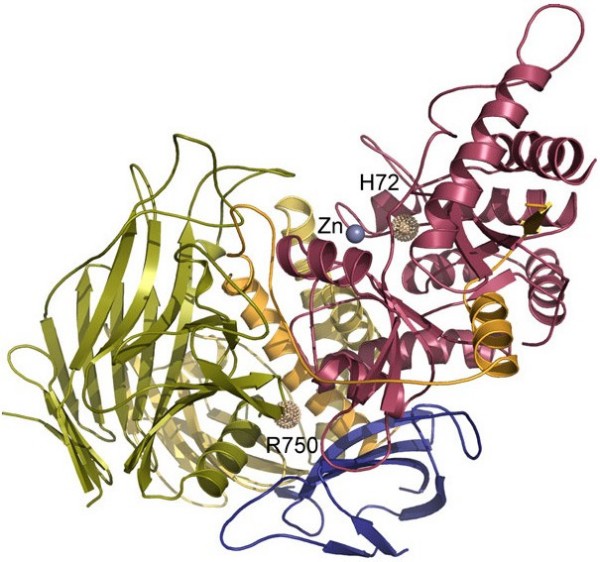
**The 3-dimensional structure of lysosomal α-mannosidase**. Peptides are coloured a-red, b-orange, c-yellow, d-green and e-blue. The active site is denoted by a Zn++ ion. Two mutant sites are displayed, demonstrating the effect of mutations c.215A>T: p.H72L affecting Zn++ coordination in the actives site (group 1 mutation, see text for explanation) and the prevalent mutation c.2248C>T: p.R750W which is likely affecting peptide e-d interaction (group 2 mutation, see text for explanation). The figure was kindly provided by Dr. P. Heikinheimo, University of Helsinki, Finland. It has been prepared with the program PyMol [72].

The enzyme is synthesized as a single chain precursor that is processed into three glycopeptides of 70, 42 and 15 kDa [54]. In humans, the 70 kDa peptide is further partially proteolysed into three more peptides that are joined by disulfide bridges – constituting all together 5 peptides (a-e) [54]. **α**-mannosidase contains 11 potential N-linked glycosylation sites all of which are occupied with either high mannose or complex type oligosaccharides [54]. Based on electron microscopy, X-ray diffraction data and the structure of *D. melanogaster *Golgi II **α**-mannosidase, the structure of the bovine lysosomal **α**-mannosidase was resolved at 2.7 Å resolution [5]. **α**-mannosidase is a di-mer and the 3-dimensional structure of bovine lysosomal **α**-mannosidase (which shares 80% identity to its human counterpart) nicely defines the fold of each peptide, identifies the active site and provide clues to understand lysosomal transport and low pH activation (Fig 5). Furthermore, the 3-dimensional structure provides the basis for understanding **α**-mannosidosis at the atomic level.

### Disease-causing mutations

A total of 40 different disease-causing mutations have been reported over the last 10 years. Except for two unrelated patients from Japan and one patient of Arabian origin [13,54,55], the patients studied originate from Europe [11,15,54,56-60]. The genetic aberrations reported are scattered all over the *MAN2B1 *gene and include missense, nonsense, small and large deletions, small insertions and splice site mutations. Most mutations are private as they occur in single or in a few families only. However, missense mutation c.2248C>T resulting in the replacement of arginine with tryptophane at amino acid position 750 (p.R750W) appears to be frequent among mannosidosis patients, as it has been reported from most European populations studied, accounting for more than 30% of all disease alleles detected by Berg *et al*. (1999) [15]. Haplotyping based on 5 internal single nucleotide polymorphisms (SNP) markers showed that a common haplotype was shared by Finnish, Polish and Italian homozygotes, whereas a second haplotype, deviating at one marker, was observed in a Turkish patient [15]. These findings indicate that the frequency and wide geographical distribution of the "p.750W" allele may result both from founder effects and from recurrent mutational events. However, further analysis including patients of other origins must be carried out in order to explore this fully.

Altogether 12 missense mutations have been functionally characterized by expression analysis in mammalian cell lines followed by enzyme activity measurements and modeling into the MAN2B1 3-dimensional structure. Missense mutations affect residues located in the active site, in the dimer interphase as well as in the interior of the protein [5,58,59,61]. Based on pulse chase experiments and immune fluorescence microscopy, Hansen *et al. *(2004) monitored the intracellular transport of mutant enzymes. They concluded that mutant enzymes (missense) could be divided into two groups: Group 1 that was correctly sorted to the lysosomes and group 2 that was retained (transport arrested) in the endoplasmic reticulum (ER) [61].

### Genotype phenotype relationships

There is no apparent correlation between *MAN2B1 *genotype and clinical phenotype in **α**-mannosidosis [15]. Clinical variation within sib ships has been observed [17,28,35,36], and patients characterized as less severely or moderately affected were shown to be homozygous for null mutations [15]. However, attempts to predict genotype/phenotype relationships often suffer from lack of data. Case reports on clinical findings are few and often include few patients and variability of methods used. Likewise, mutation reports often lack sufficient clinical data.

Enzyme activity measurements from patient leukocytes or fibroblast cells provide no clue either to a *MAN2B1 *genotype/phenotype correlation as these values range from 5% to 15% of normal levels. Using a refined method Berg *et al. *(1999) demonstrated that the level of cross-reacting **α**-mannosidase activity was less than 1.3% of that in controls, with no consistent variation among affected individuals. However, one cannot rule out the existence of *MAN2B1 *genotypes ("mild" mutations) that cause sub-clinical symptoms associated with residual enzyme activity. Such cases would likely escape detection as they will not be severe enough to arouse the suspicion of **α**-mannosidosis.

Environmental factors and other genetic factors may contribute to the clinical heterogeneity seen in **α**-mannosidosis. Environmental factors might be exposure to pathogens causing recurrent infections and thereby worsening of the disease symptoms. Other genetic factors might be those that encode other mannosidases like cytosolic **α**-mannosidase that potentially might contribute to the intra cellular clearance of undigested oligosaccharides [14,41,62-64]. However, no such salvage pathway has yet been proven to contribute to the clinical variability.

A thorough clinical and molecular investigation of many patients is warranted in order to explore the clinical variability of **α**-mannosidosis. Indeed, a study on the natural history of mannosidosis was recently initiated by HUE-MAN – a multinational research project supported by the sixth framework program of the European Union [8].

## Diagnostic methods

### Peripheral blood examination

Light microscopy or transmission electron microscopy (TEM) demonstrates vacuoles in bone marrow smears and lymphocytes from peripheral blood in most affected individuals (Fig 4) (reviewed in [14]). Although detection of vacuoles by microscopy is a useful screening test, supplementary investigations are necessary when α-mannosidosis is suspected.

### Oligosaccharides in urine

Elevated urinary excretion of mannose-rich oligosaccharides can be demonstrated by thin-layer chromatography [41] or high performance liquid chromatography (HPLC) [65]. This finding is suggestive of **α**-mannosidosis, but not diagnostic.

### Acid α-mannosidase activity

The most efficient and reliable method of establishing the diagnosis of **α**-mannosidosis is the assay of acidic **α**-mannosidase activity in leukocytes or other nucleated cells. This fluorometric assay is performed at low pH (usually at pH 4) with the substrate 4-methylumbelliferyl **α**-D-mannopyranoside. In affected individuals, acid **α**-mannosidase enzyme activity in peripheral blood leukocytes is 5%–15% of normal activity. Residual enzyme activity could possibly represent **α**-mannosidase activity from other organelles or compartments (*e.g.*, Golgi apparatus; MAN2A1, cytosol; MAN2C1 or ER; MAN1B1), showing some activity also at low pH. Following immunoprecipitation with anti-acid **α**-mannosidase polyclonal antibodies, acid **α**-mannosidase enzyme activity ranges from 0.1% to 1.3% of normal [15]. Such testing is not performed routinely. In carriers, acid **α**-mannosidase enzyme activity is usually 40%–60% of normal, and is therefore unreliable for carrier detection because of the overlap between carriers and non-carriers.

### Genetic testing

Identification of disease causing mutations is carried out on DNA from peripheral blood cells, by polymerase chain reaction (PCR) amplification of all 24 *MAN2B1 *exons followed by DNA sequencing.

### Differential diagnosis

The main symptoms of mannosidosis, like dysmorphic traits, dysostosis, and mental retardation, are shared with the symptoms in many lysosomal storage diseases like mucopolysaccharidosis.

## Genetic counseling

According to autosomal recessive inheritance, offspring of carrier parents have 25% risk of being affected whereas 50% will be non-symptomatic carriers.

## Antenatal diagnosis

Prenatal testing is available for pregnancies of carrier parents. Prenatal testing may be performed by analysis of acid **α**-mannosidase enzyme activity in fetal cells obtained by chorionic villus sampling at 10–12 weeks gestation or by amniocentesis at 15–18 weeks. DNA from the same sources can be used for mutation analysis. Preferably, mutation analysis should be carried out in the parents in advance of pregnancy. Genotype does not allow prediction of severity of disease.

## Management

### Non-specific management

In general, the approach to the patients should be pro-active, searching for emerging complications. After a full physical examination, focusing on the known complications of mannosidosis like hydrocephalus, otitis media, hearing loss, dental state, joint status, kyphoscoliosis, and mental state, a plan should be made to limit the health consequences for the patient.

This would also include examination by an ophthalmologist, an otolaryngologist, audiometry and neuropsychological assessment, blood tests, and skeletal evaluation with radiographs, especially of the head, spine, knees or other skeletal sites displaying symptoms.

#### Hydrocephalus

Early diagnosis with measurement of head circumference, diagnosis with ultrasound, skull radiographs or computed tomography (CT) [20]. Ventriculocaval shunt should be preferred before ventriculoperitoneal shunt due to reduced ability of resorption in the peritoneal cavity in mannosidosis (Malm D: personal observation).

#### Otitis media

Diagnosis is simple and insertion of pressure-equalizing tubes will reduce the impact of the mechanical component of reduced hearing. There is a variety of tubes, and tubes with long-term stability should be preferred, since the condition is usually long lasting [29].

#### Hearing

Diagnosis is made with audiometry in cooperating patients, but can be difficult in young children and severely retarded patients. In some cases the brainstem auditory evoked response (BAER) test measuring brain wave activity that occurs in response to clicks or certain tones, can be useful. For speech therapy to be effective, hearing aids should be provided.

#### Dental state

Caries seem frequent because of reduced dental quality combined with tooth grinding or acid reflux from the stomach. Regular dental support and good dental hygiene is obviously important.

#### Joint status

Goniometry is used in the measurement of joint pathology. Kyphoscoliosis is measured according to Cobb, and can be corrected with orthopedic surgery (Fig 2). Genu valgum deformity can be treated with epiphyseal stapling in growing children, but must be performed early to be effective [23].

#### Mental state

Can be monitored with various tests, such as Wechsler. Nonverbal tests can compensate for the hearing deficit.

#### Specific treatment

In **α**-mannosidosis all cells are devoid of **α**-mannosidase activity. Early observations showed that cells producing **α**-mannosidase were able to transfer the enzyme to mannosidosis cells [63,66]. The rationale for bone marrow transplantation (BMT) in mannosidosis is that enzyme-producing donor cells repopulate the host tissues and transfer enzyme to nearby enzyme-deficient host cells.

In 1987 Will *et al. *performed BMT in a patient with **α**-mannosidosis. However, the patient died 18 weeks after successful grafting due to procedure related complications. The *post mortem *examination showed that transplantation reversed the somatic changes of **α**-mannosidosis, but did not affect lysosomal storage within brain tissue. It was therefore concluded that BMT may not be a suitable treatment for **α**-mannosidosis [67]. However, the findings of nil effect in the brain could be explained by the only 50% activity in donor cells which came from the mother (being a carrier of alpha-mannosidosis), the treatment of prednisone which could have influenced synthesis of protein, or the short observation period. Because of the blood-brain barrier, the main question remained whether BMT could improve the pathology of the central nervous system. In 1994, Steven Walkley reported that an early BMT could prevent neurological deterioration in a cat model [68]. A possible explanation of the neuronal benefits of BMT could be migration of donor-derived cells to the CNS of the recipient [69]. Later, Wall *et al. *(1998) presented a single case with BMT, claiming complete resolution of the recurrent infectious disease and organomegaly, improvement in the bone disease, and stabilization of neurocognitive function during a 2 year observation period [70].

Subsequently, a number of unpublished BMTs were performed, and in 2004, Grewal *et al. *could present results from four patients, aged 3 to 23 years, that had undergone BMT [69]. In short, they claimed that intellectual function stabilized, with improvement in adaptive skills and verbal memory function in 3 of 4 patients. Especially hearing improved to normal or near normal, but for speech frequencies only.

The possible benefits of BMT must be weighed against the overall risk of procedure related morbidity and mortality. The benefits are greater in younger patients before complications have developed, and also transplant related complications are more frequent and severe in older patients. Therefore, BMT is an option in the first decade of life which makes early identification of affected patients critical.

## Prognosis

The long-term prognosis is poor. There is an insidiously slow progression of neuromuscular and skeletal deterioration over several decades, making most patients wheel-chair dependent. No patients manage to be completely socially independent. Many patients are over 50 years of age.

## Unresolved questions

Enzyme replacement therapy (ERT) is a therapeutic modality in other lysosomal storage diseases, like Gaucher, Fabry or Pompe disease. In **α**-mannosidosis, experiments with ERT have been performed in an artificial knock-out mouse model [45], and in a naturally occurring guinea pig model [71]. The reduction in storage material was evident in almost all tissues in both models. However, whereas the first study found a reduction of mannose containing oligosaccharides in the brain to less than 30% of that in control mice with **α**-mannosidosis [45], a similar cerebral improvement was not found in guinea-pigs [71]. The development of ERT in human is the long term objective of the European HUE-MAN project [8].

## Abbreviations

Central nervous system: CNS; bone marrow transplantation: BMT; intelligence quotient: IQ; interleukin-2: IL-2; systemic lupus erythematosus: SLE; single nucleotide polymorphisms: SNP; endoplasmic reticulum: ER; polymerase chain reaction: PCR; computed tomography: CT; brainstem auditory evoked response: BAER; Enzyme replacement therapy: ERT.

## Competing interests

The authors declare that they have no competing interests.

## Authors' contributions

The authors equally contributed to this review article. They read and approved the final version of the manuscript.

## Consent

Written consent for publication of photographs was obtained from the patients or legal guardians where required.
